# Endogenous retrovirus activation: potential for immunology and clinical applications

**DOI:** 10.1093/nsr/nwae034

**Published:** 2024-01-25

**Authors:** Jundan Yu, Peishan Qiu, Jingwen Ai, Bo Liu, Guan-Zhu Han, Fan Zhu, Wenhong Zhang, Jie Cui

**Affiliations:** Department of Infectious Diseases, Shanghai Key Laboratory of Infectious Diseases and Biosafety Emergency Response, National Medical Center for Infectious Diseases, Huashan Hospital, Shanghai Medical College, Fudan University, China; CAS Key Laboratory of Molecular Virology & Immunology, Shanghai Institute of Immunity and Infection, Chinese Academy of Sciences, China; University of Chinese Academy of Sciences, China; CAS Key Laboratory of Molecular Virology & Immunology, Shanghai Institute of Immunity and Infection, Chinese Academy of Sciences, China; University of Chinese Academy of Sciences, China; Department of Infectious Diseases, Shanghai Key Laboratory of Infectious Diseases and Biosafety Emergency Response, National Medical Center for Infectious Diseases, Huashan Hospital, Shanghai Medical College, Fudan University, China; Shanghai Huashen Institute of Microbes and Infections, China; CAS Key Laboratory of Molecular Virology & Immunology, Shanghai Institute of Immunity and Infection, Chinese Academy of Sciences, China; University of Chinese Academy of Sciences, China; College of Life Sciences, Nanjing Normal University, China; State Key Laboratory of Virology and Department of Medical Microbiology, School of Basic Medical Sciences, Wuhan University, China; Department of Infectious Diseases, Shanghai Key Laboratory of Infectious Diseases and Biosafety Emergency Response, National Medical Center for Infectious Diseases, Huashan Hospital, Shanghai Medical College, Fudan University, China; Shanghai Huashen Institute of Microbes and Infections, China; The Institute of Infection and Health Research, Fudan University, China; Key Laboratory of Medical Molecular Virology (MOE/MOH), Shanghai Medical College, Fudan University, China; Department of Infectious Diseases, Shanghai Key Laboratory of Infectious Diseases and Biosafety Emergency Response, National Medical Center for Infectious Diseases, Huashan Hospital, Shanghai Medical College, Fudan University, China; CAS Key Laboratory of Molecular Virology & Immunology, Shanghai Institute of Immunity and Infection, Chinese Academy of Sciences, China; University of Chinese Academy of Sciences, China; Shanghai Huashen Institute of Microbes and Infections, China; The Institute of Infection and Health Research, Fudan University, China; Laboratory for Marine Biology and Biotechnology, Qingdao Marine Science and Technology Center, China

Endogenous retroviruses (ERVs), the remnants of ancient retrovirus infections, constitute approximately 8% of the human genome and play important roles in human evolution, development, and diseases. Multiple drugs, such as DNA methyltransferase inhibitors and lysine-specific histone demethylase 1 inhibitors, reactivate ERVs, induce viral mimicry and trigger innate immune responses to enhance tumor immunogenicity. Derepressed ERVs containing open reading frames can be translated into tumor-specific antigens and presented on the cancer cell surface, where they are recognized by T cells and B cells that initiate adaptive immune responses. The development of an mRNA delivery system based on ERV-derived proteins, as well as tumor-specific expression vectors using dual-function regulatory elements of ERVs, further enrich the repertoire of vectors useful for clinical therapy. Here, we discuss the clinical and immunological applications of ERVs at the DNA, RNA, and protein levels.

**Figure 1.**
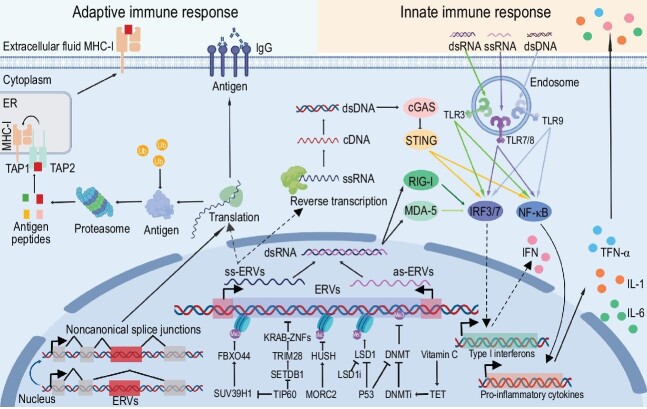
Regulation of ERVs and ERV-mediated innate and adaptive immune responses. The host silences ERVs by DNA methylation and histone modification using a variety of proteins, such as TRIM28, LSD1, DNMT and so on. Reactivated ERVs can form ssRNA, dsRNA and dsDNA, which are recognized by RIG-I, MDA-5, cGAS and TLRs, all of which induce the expression of type I interferons and pro-inflammatory cytokines, such as TNF-alpha, IL-1, IL-6, to trigger innate immune responses. Derepressed ERVs can be translated into neoantigens that are presented on the cell surface by MHC-I and recognized by CD8^+^ T cells to engage adaptive immune responses. ERVs can also produce tumor-specific antigens by noncanonical splice junctions between exons and ERVs. Some proteins, especially envelope proteins, can also be presented on the cell surface as antigens and recognized by antibodies secreted by B cells and could serve as potential targets for immunotherapy.

Retroviruses infect a wide range of vertebrate hosts and may integrate into the genome of host cells, including somatic cells and germline cells, to form proviruses via reverse transcription of RNA into double-stranded DNA (dsDNA). Germ cells occasionally integrate into proviruses that may develop offspring and form endogenous retroviruses (ERVs), which constitute approximately 8% of the genomes of human populations and are vertically transmitted in a Mendelian fashion [1]. Full-length ERVs consist of two long terminal repeats (LTRs) flanking both the 5′- and 3′-termini of the ERV-internal sequence, which contains four coding genes: *gag, pro, pol* and *env*. In general, ERV production is strictly regulated by various host proteins such as tripartite motif-containing 28 (TRIM28), DNA methyltransferase (DNMT), lysine-specific histone demethylase 1 (LSD1), HUSH complex, histone regulator A (HIRA), SWI-SNF complex, microrchidia (MORC) proteins, heterochromatin protein 1 (HP1), death-domain-associated protein (DAXX), and ATP-dependent helicase (ATRX) through DNA methylation and histone modifications, such as H3K9me3 and H3K27me3 (Fig. 1). The production of RNA, such as PIWI-interacting RNA (piRNA), long noncoding RNA (lncRNA), and double-stranded RNA (dsRNA), derived from activated ERVs is also mediated by the PIWI protein, nuclear exosome targeting (NEXT) complex or METTL3-METTL14 complex [2].

ERVs are reactivated during embryonic development, cell differentiation, aging, neurodegenerative disease onset, tumorigenesis, viral infection, radiotherapy, and epigenetic drug therapy, and exhibit obvious cell-type specificity [3]. Both RNA and DNA, such as single-stranded RNA (ssRNA), dsRNA and dsDNA, produced by abnormally active ERVs enriched H3K27ac can act as pathogen-associated molecular patterns (PAMPs) and can be recognized by cytoplasmic nucleic acid sensors, such as retinoic acid-inducible gene I (RIG-I), melanoma differentiation-associated protein 5 (MDA-5), Toll-like receptors (TLRs) and cyclic GMP-AMP synthase (cGAS), which serve as specific pattern recognition receptors (PRRs), to trigger innate immune responses through mimicking those of exogenous viruses [4]. This process is known as viral mimicry, which can inhibit viral infection, increase the immunogenicity of tumor cells, and remodel the tumor microenvironment to favor T-cell infiltration. For example, the DNA methyltransferase inhibitor (DNMTi) 5-azacytidine and the CDK4/6 inhibitor abemaciclib, which are used in clinical antitumor therapy, can induce ERV reactivation through DNA demethylation and dsRNA-mediated viral mimicry. The LSD1 inhibitor dinitroazetidine derivative RRx-001 and the CDK9 inhibitor MC180295, which showed antitumor activity in clinical trials, derepress ERVs to form dsRNA and trigger type I interferon pathway activation. Both vitamin C, which upregulates ten-eleven translocation (TET) enzyme activity to promote DNA demethylation, and the histone methyltransferase inhibitor UNC0638 combined with DNMTi show synergistic antitumor effects. DNMTi 5-azacytidine in combination with other types of cancer drugs, such as romidepsin, gemcitabine, venetoclax, LSD1 inhibitor, temozolomide, and isocitrate dehydrogenase-1 (IDH1) inhibitor, increases the treatment effect, indicating that the key molecules regulating ERVs are expected to become tumor treatment targets and may even be used in combination with existing anticancer drugs (Fig. 1).

Most ERVs have lost coding capacity over time. However, a subset of ERVs containing open reading frames can be translated into proteins or peptides that have been co-opted to serve host functions, including syncytin-1 and syncytin-2, which drive cell-cell fusion and form syncytia in placental development. Syncytin-1 and syncytin-2 which contain immunosuppressive domains can reduce the production of the T-helper 1 cytokines such as TNF-α and IFN-γ. In addition, *env*-derived suppressyn and Fv-4, *gag*-derived Fv-1, can restrict retroviral infection by binding to and competing for cell surface receptors or disrupting the assembly of the viral capsid core. Abnormally activated ERVs encode tumor-specific antigens (TSAs) due to dysregulated epigenetic modifications that trigger adaptive immune responses in tumor cells. In addition, noncanonical splice junctions between exons and ERVs are sources of neoantigens because ERVs can provide alternative splice sites (Fig. 1) [5]. TSAs are degraded by the proteasome and are presented on the tumor cell surface through major histocompatibility class I (MHC-I) and recognized by CD8^+^ T cells [6]. Some other ERV-derived proteins can be transported to cell membranes and promote immunotherapy as antigens that bind to antibodies secreted by B cells. ERV derepression exerts a synergistic effect with immune checkpoint blockers, such as programmed death-ligand 1 (PD-L1) and cytotoxic T-lymphocyte associated antigen 4 (CTLA4) blockers [7]. ERVs are even involved in the formation of HERV-H LTR-associating protein 2 (HHLA2), which is an attractive target for immunotherapy. HHLA2 is aberrantly expressed in solid tumors with even higher PD-L1 expression and depends on corresponding receptors to exert costimulatory or coinhibitory effects. Treatment of tumors with DNMTi promotes the production of ERV-derived TSAs and does not affect the recognition of antigens by T cells. In addition, reactivated ERVs trigger innate immune responses and then induce the expression of type I interferons, which increases the expression of genes associated with MHC-I to promote antigen presentation on the surface of tumor cells. These results suggest that innate and adaptive immunity are induced by derepressed ERVs and exert synergistic effects, showing great prospects for clinical application. On January 24, 2024, the Evaxion Biotech, a clinical-stage AI-immunology platform company, announced that it will develop ERV cancer vaccines, which may broaden the applicability of cancer vaccines.

PEG10, a Gag homolog derived from Ty3/Gypsy of LTR-retrotransposons, can bind to its own mRNA to form virus-like particles (VLPs), together with syncytin, derived from the Env, can be used for the construction of an mRNA delivery system. Since PEG10 and syncytin are both expressed in the host, this delivery system may show great potential in clinical applications for its lower immunogenicity and higher efficiency compared to mRNA delivery methods by lipids, polymeric nanoparticles or electroporation. However, the potential side effects, packaging capabilities, and the feasibility of large-scale production regarding the mRNA delivery system are currently unclear. Both Ty3/Gypsy and ERVs of LTR retrotransposons contain core capsid domain of Gag that binds to their own RNA to form VLPs. The Gag and Env of ERVs may also serve as a candidate library for future development and optimization of the mRNA delivery system (Fig. 1) [8].

ERVs are tightly regulated by the hosts, but ERVs are extensively involved in the formation of *cis*-regulatory elements such as promoters, enhancers, silencers and insulators to regulate the expression of host genes and show significant cell-type specificity [9]. For example, LTR7 is activated by binding of transcription factors (TFs) specifically expressed in stem cells; these TFs include OCT4, NANOG, SOX2, and LBP9, which show obvious cell type-specific promoter activity. Some ERV-derived silencers were identified as enhancers in other cell types, suggesting that ERVs can be used as dual-function regulatory elements, which can act as enhancers in some cellular contexts and as silencers in others. Tumor-specific expression vectors, such as oncolytic virus vectors, can be constructed using tumor-specific *cis*-regulatory elements derived from ERVs that are repressed in normal cells to specifically express effector genes in tumors.

In summary, the reactivation of ERVs can induce viral mimicry to trigger innate immune responses, be translated into neoantigens to induce adaptive immune responses, and may be used to develop mRNA delivery systems and tumor-specific expression vectors for immunotherapy. The ERVs abundant in the genomes show great prospects for clinical and immunological applications.
